# Mechanisms that Link Chronological Aging to Cellular Quiescence in Budding Yeast

**DOI:** 10.3390/ijms21134717

**Published:** 2020-07-02

**Authors:** Karamat Mohammad, Jennifer Anne Baratang Junio, Tala Tafakori, Emmanuel Orfanos, Vladimir I. Titorenko

**Affiliations:** Department of Biology, Concordia University, Montreal, QC H4B 1R6, Canada; karamat.mohammad@concordia.ca (K.M.); jennifer.anne.junio@gmail.com (J.A.B.J.); tala.t@live.ca (T.T.); orfanosemmanuel@gmail.com (E.O.)

**Keywords:** yeast, cellular quiescence, cellular aging, cell cycle, properties of quiescent yeast, quiescence entry, quiescence maintenance, metabolism, caloric restriction, yeast chronological aging

## Abstract

After *Saccharomyces cerevisiae* cells cultured in a medium with glucose consume glucose, the sub-populations of quiescent and non-quiescent cells develop in the budding yeast culture. An age-related chronology of quiescent and non-quiescent yeast cells within this culture is discussed here. We also describe various hallmarks of quiescent and non-quiescent yeast cells. A complex aging-associated program underlies cellular quiescence in budding yeast. This quiescence program includes a cascade of consecutive cellular events orchestrated by an intricate signaling network. We examine here how caloric restriction, a low-calorie diet that extends lifespan and healthspan in yeast and other eukaryotes, influences the cellular quiescence program in *S. cerevisiae*. One of the main objectives of this review is to stimulate an exploration of the mechanisms that link cellular quiescence to chronological aging of budding yeast. Yeast chronological aging is defined by the length of time during which a yeast cell remains viable after its growth and division are arrested, and it becomes quiescent. We propose a hypothesis on how caloric restriction can slow chronological aging of *S. cerevisiae* by altering the chronology and properties of quiescent cells. Our hypothesis posits that caloric restriction delays yeast chronological aging by targeting four different processes within quiescent cells.

## 1. Introduction

When *Saccharomyces cerevisiae* cells are cultured under aerobic conditions in a nutrient-rich liquid medium with 2% glucose, they are not limited in calorie supply [1,2,3,4,5,6]. They exist under so-called non-caloric restriction (non-CR) conditions [1,2,3,4,5,6]. After these cells consume glucose as a sole exogenous carbon source, they enter a diauxic shift period [3,4]. At the time of the diauxic shift, yeast cells decelerate the growth and switch the mode of their metabolism from aerobic alcoholic fermentation to aerobic ethanol catabolism and mitochondrial respiration [3,4]. During the diauxic shift, some *S. cerevisiae* cells in the culture arrest their cell-division cycle at the nutrient-dependent checkpoint “START A” in the late G_1_ phase [7,8,9,10,11]. At the time of such cell cycle arrest, the budding yeast culture begins to accumulate the sub-populations of quiescent (Q) and non-quiescent (NQ) cells [7,8,9,10,11]. The Q and NQ cells in yeast cultures under non-CR conditions differ from each other in physical, morphological, reproductive, biochemical, and physiological properties [7,8,9,10,11]. A signaling network that integrates a distinct set of the nutrient-sensing signaling pathways and protein kinases orchestrates the development of properties characteristic of Q cells [4,9]. After *S. cerevisiae* cells cultured under non-CR conditions consume ethanol as a carbon source, they enter the stationary (ST) phase of culturing and the process of their chronological aging begins [3,4,5,6]. The chronological aging of *S. cerevisiae* is assessed by measuring the percentage of yeast cells that in liquid cultures remain viable at different time points following the entry of a cell population into the non-proliferative ST phase of culturing [5,6]. Here, we compare the properties of Q and NQ cells cultured under non-CR conditions. Noteworthy, the pace of yeast chronological aging and the longevity of chronologically aging yeast under non-CR conditions depend on the cell entry into and advancement through a quiescence program. *S. cerevisiae* cells that are not limited in calorie supply enter this cellular quiescence program during the diauxic shift and advance through it during the ST phase of culturing [7,8,9,10,11,12]. As any programmed biological event, this cellular quiescence program (1) is a genetically defined, regulated process, (2) can be accelerated or decelerated by genetic manipulations that alter the abundancies and/or activities of only specific proteins, (3) integrates a cascade of consecutive cellular events that follow each other in a particular order and are regulated by a specific signaling network, (4) is initiated in response to certain stimuli (e.g., nutrient deprivation or chronological aging), and (5) provides a particular benefit for the development, survival, and/or stress resistance of a cell population [7,8,9,10,11,12,13,14,15,16,17].

The chronological aging of *S. cerevisiae* can be slowed down, and its longevity can be extended by CR [1,2], a low-calorie dietary regimen without malnutrition that prolongs lifespan and postpones the onset of age-related pathologies in other yeast species, nematodes, fruit flies, fishes, dogs, rodents, and primates [18,19,20]. The effects of CR on chronological aging of *S. cerevisiae* are usually investigated in budding yeast cultured in a nutrient-rich or nutrient-limited synthetic minimal medium initially containing 0.2% or 0.5% glucose [2,5,6]. In contrast to a nutrient-limited synthetic minimal medium, a nutrient-rich medium has plenty of amino acids, nucleotides, vitamins, and other nutrients [21,22,23]. Therefore, the use of a nutrient-rich medium with 0.2% or 0.5% glucose for chronological aging studies under CR conditions provides several important advantages as compared to the use of a minimal synthetic medium [2].

We previously purified the Q and NQ cell populations from budding yeast cultured in a nutrient-rich medium under CR or non-CR conditions [24,25]. We recovered these cell populations at different stages of the chronological aging process and compared their properties [24,25]. Here, we discuss how CR slows the conversion of Q cells into NQ cells because this low-calorie diet alters the specific properties of Q cells. We also examine the evidence that the ability of CR to alter these properties of Q cells is responsible for the CR-dependent delay of chronological aging in budding yeast.

## 2. Traits of Q and NQ Cells Found in Yeast Populations Cultured Under Non-CR Conditions

**Figure 1:**
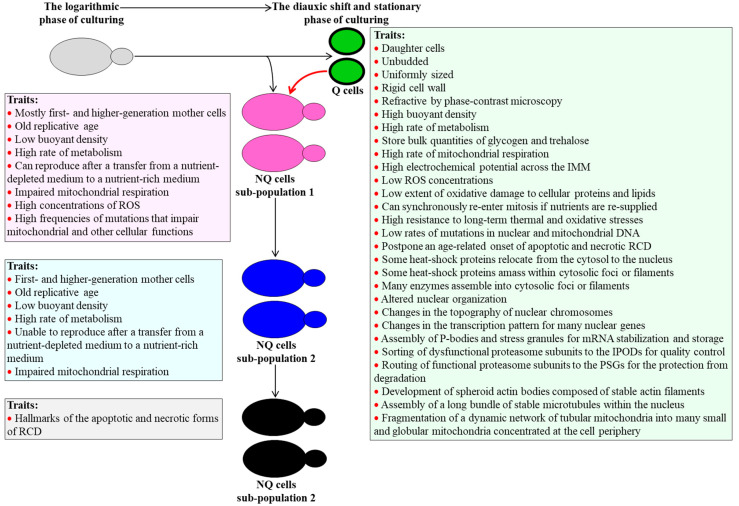
At the time of the diauxic shift, a *Saccharomyces cerevisiae* culture under non-caloric restriction (CR) conditions begins to accumulate a sub-population of quiescent (Q) cells and a sub-population 1 of non-quiescent (NQ) cells. NQ cells of the sub-population 1 undergo a stepwise conversion into NQ cells of the sub-population 3. The Q and NQ cell sub-populations differ from each other in many distinct traits. In chronologically aging *S. cerevisiae* cultures, Q cells undergo conversion into NQ cells of the sub-population 1 in an age-related manner. See the text for more details. Abbreviations: IMM, the inner mitochondrial membrane; IPODs, insoluble protein deposits; PSGs, proteasome storage granules; RCD, regulated cell death; ROS, reactive oxygen species.

Those cells in a budding yeast population cultured under non-CR conditions that undergo cell-cycle arrest enter a non-proliferative state called G_0_ [7,8,9,10,11,12]. They form the Q cell sub-population [7,8,9,10,11,12]. In contrast, those cells in the budding yeast population not limited in calorie supply that does not arrest their cell cycle give rise to at least three sub-populations of NQ cells [7,8,9,10,11,12]. The Q and NQ cell sub-populations differ from each other in many traits. These traits are discussed below and schematically depicted in Figure 1.

### 2.1. Traits of Q Cells under Non-CR Conditions

Q cells of *S. cerevisiae* cultured under non-CR conditions are mostly unbudded and uniformly sized daughter cells that are surrounded by a thick cell wall, refract light when viewed through a phase-contrast microscope, and have a high buoyant density (Figure 1) [7,8,10,11,12,24]. These cells display a high rate of metabolism, amass glycogen and trehalose, have actively respiring mitochondria capable of maintaining a high electrochemical potential across the inner membrane, exhibit low concentrations of reactive oxygen species (ROS), and sustain a low extent of oxidative damage to cellular proteins and lipids (Figure 1) [7,8,9,10,11,12,24]. Q cells of *S. cerevisiae* cultured under non-CR conditions can synchronously re-enter mitosis and reproduce after being transferred from a nutrient-depleted liquid medium to a surface of a nutrient-rich solid medium or a fresh liquid medium (Figure 1) [7,8,10,11,12,24]. These cells exhibit low susceptibility to chronic (long-term) thermal and oxidative stresses, rarely undergo mutations that affect mitochondrial and other cellular functions, and postpone an age-related onset of the apoptotic and necrotic forms of regulated cell death (RCD) (Figure 1) [7,8,9,10,11,12,24].

Recent findings revealed many other traits characteristic of Q cells in *S. cerevisiae* cultures not limited in calorie supply. Because these traits have been comprehensively discussed elsewhere [17], we outline them below only briefly. Q cells of budding yeast cultured under non-CR conditions have some heat-shock proteins relocated from the cytosol to the nucleus; they amass other heat-shock proteins and many metabolic and non-metabolic enzymes within multiple cytosolic foci or filaments (Figure 1) [17,26,27,28,29,30,31,32]. These cells also display altered nuclear organization, global changes in the topography of nuclear chromosomes, and substantial alterations in the transcription pattern for many nuclear genes (Figure 1) [17,33,34,35,36,37,38,39,40,41]. Q cells of *S. cerevisiae* under non-CR conditions enhance their ability to sustain cellular proteostasis by employing the following three mechanisms: (1) by assembling P-bodies and stress granules for mRNA stabilization and storage (respectively) in the cytosol (Figure 1) [32,42,43,44], (2) by routing dysfunctional proteasome subunits from the nucleus to the so-called insoluble protein deposits (IPODs) for quality control (Figure 1) [45], and (3) by sorting functional proteasome subunits from the nucleus to the so-called proteasome storage granules (PSGs) for the protection from degradation (Figure 1) [46,47,48,49]. Q cells of budding yeast under non-CR conditions re-arrange their cytoskeleton. These cells dismantle an elaborate network of dynamic actin cables and develop spheroid actin bodies, which consist of stable actin filaments (Figure 1) [50]. Q cells also disassemble a cytosolic array of dynamic microtubules and build a long bundle of stable microtubules in the nucleus (Figure 1) [34]. Q cells of *S. cerevisiae* under non-CR conditions undergo a fragmentation of their dynamic network of tubular mitochondria into many small and globular mitochondria concentrated at the cell periphery (Figure 1) [51].

Although mechanisms underlying the regulated establishment of many traits characteristic of Q cells under non-CR conditions remain unknown, some of these traits are essential contributors to the abilities of Q cells to exit quiescence, re-enter the cell cycle, and resume proliferation [9,10,11,12,17,24,26,29,34,47,48,49,50]. Moreover, some of these traits (but not all of them) also contribute to longevity assurance in chronologically aging budding yeast [9,10,11,12,24,34,39,47,48,49,50]. Mechanisms that link these essential traits of Q cells to their exit from quiescence, proliferation, and chronological aging are presently unexplored. 

### 2.2. Traits of NQ Cells under Non-CR Conditions

Three distinct sub-populations of NQ cells exist in an *S. cerevisiae* culture under non-CR conditions [7,8,9,10,11,12].

Most NQ cells present in the sub-population 1 are first- and higher-generation mother cells, although some daughter cells can also be found there (Figure 1) [7,8,9,10,11,12]. Most NQ cells in the sub-population 1 have one or more bud scars on their surface [7,8,9,10,11,12]. Because each bud scar is formed on the surface of a mother cell when it buds off a new daughter cell [52,53,54], the NQ cells in the sub-population 1 are mainly replicatively older than the daughter Q cells (Figure 1) [7,8,9,10,11,12,24,55,56,57]. Akin to Q cells, the NQ cells in the sub-population 1 are metabolically active (Figure 1) [7,8,9,10,11,12,24]. These NQ cells can reproduce after being transferred from a nutrient-depleted medium to a nutrient-rich medium (Figure 1) [7,8,9,10,11,12,24]. Unlike Q cells, the NQ cells in the sub-population 1 have a low buoyant density and display impaired mitochondrial respiration, high concentrations of ROS, and elevated frequencies of mutations that impair mitochondrial and other cellular functions (Figure 1) [7,8,9,10,11,12,24].

NQ cells in the sub-population 2 are first- and higher-generation mother cells of low buoyant density that are replicatively old (Figure 1) [7,8,9,10,11,12]. These NQ cells exhibit a high rate of metabolism, like NQ cells in sub-population 1 (Figure 1) [7,8,9,10,11,12]. Unlike NQ cells in the sub-population 1, NQ cells in the sub-population 2 cannot reproduce if transferred from a nutrient-depleted medium to a nutrient-rich medium (Figure 1) [7,8,9,10,11,12]. It is conceivable, therefore, that NQ cells in the sub-population 2 may originate from NQ cells in the sub-population 1 (Figure 1) [7,8,9,10,11,12].

Most NQ cells in the sub-population 3 are reproductively incompetent cells displaying the characteristic traits of the apoptotic and necrotic forms of RCD (Figure 1) [7,8,9,10,11,12]. These cells are believed to be descendants of NQ cells in sub-population 2 (Figure 1) [7,8,9,10,11,12].

It is feasible that in chronologically aging *S. cerevisiae* cultures under non-CR conditions the pathway of a stepwise transformation of NQ cells in the sub-population 1 into NQ cells in the sub-population 3 is sustained via an age-related conversion of Q cells into NQ cells that compose the sub-population 1 (Figure 1, red arrow) [7,8,9,10,11,12,24]. Mechanisms regulating such age-related conversion of Q cells of the sub-population 1 of NQ cells remain unknown.

## 3. CR Diet Alters an Age-Related Chronology and Properties of Q and NQ Cells Found in Yeast Populations

**Figure 2:**
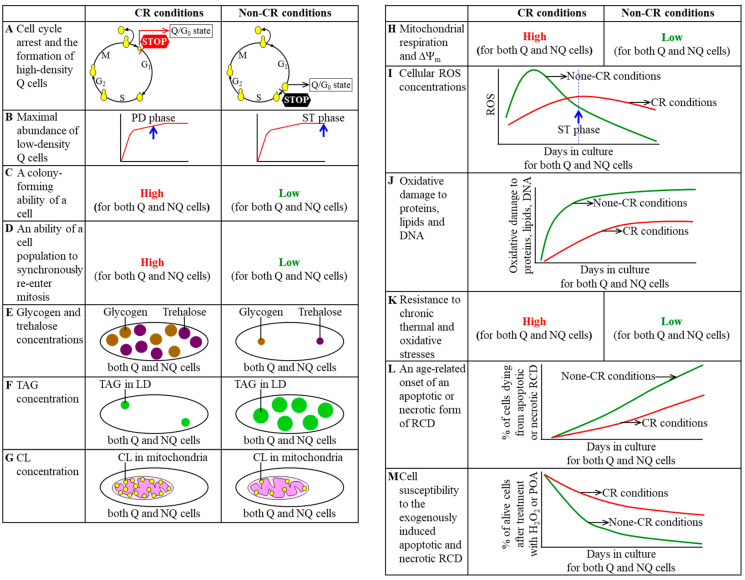
The caloric restriction (CR) diet alters an age-related chronology and specific traits of quiescent (Q) and non-quiescent (NQ) cell sub-populations. These aspects of an age-related chronology and these specific traits are named in different panels of the figure. They include the following: cell cycle arrest and the formation of high-density Q cells (**A**), maximal abundance of low-density Q cells (**B**), a colony-forming ability of a cell (**C**), an ability of a cell population to synchronously re-enter mitosis (**D**), glycogen and trehalose concentrations (**E**), triacylglycerol (TAG) concentration (**F**), cardiolipin (CL) concentration (**G**), mitochondrial respiration and the electrochemical potential across the inner mitochondrial membrane (ΔΨm) (**H**), cellular reactive oxygen species (ROS) concentrations (**I**), oxidative damage to proteins, lipids and DNA (**J**), resistance to chronic thermal and oxidative stresses (**K**), an age-related onset of an apoptotic or necrotic form of regulated cell death (RCD) (**L**) and cell susceptibility to the exogenously induced apoptotic and necrotic RCD (**M**). See the text for more details. Other abbreviations: CL, cardiolipins; LD, lipid droplets; POA, palmitoleic acid.

In a recent study, the Q and NQ cell sub-populations were purified from differently aged *S. cerevisiae* populations cultured in a nutrient-rich medium under CR or non-CR conditions [24]. A comparative analysis of these cell sub-populations has shown that the CR diet revises an age-related chronology of Q and NQ cells and alters several traits characteristic of these cells [24]. The effects of CR on the chronology and traits of Q and NQ cells are discussed below and schematically depicted in Figure 2.

CR creates a sub-population of high-density Q cells by arresting the cell-division cycle at a different checkpoint in the G_1_ phase than the one responsible for the formation of high-density Q cells under non-CR conditions [24,25]. After cells consume glucose under non-CR conditions, the formation of the high-density Q cell sub-population and entry into the G_0_ state occur in late G_1_ [7,8,9,10,11]. In contrast, after cells consume glucose under CR conditions, the formation of the high-density Q cell sub-population and entry into the G_0_ state happen at a checkpoint in early G_1_ (Figure 2A) [24]. 

CR accelerates an age-related buildup of low-density Q cell sub-population in chronologically aging yeast cultures [24,25]. Indeed, the abundance of low-density Q cells in non-CR yeast cultures reaches a plateau in the ST growth phase, while the abundance of low-density Q cells in CR yeast cultures attains a steady-state level already in the post-diauxic (PD) growth phase (Figure 2B) [24]. 

Both Q and NQ cells formed under CR conditions retain their abilities to reproduce for a longer period of their chronological lifespans than age-matched Q and NQ cells formed under non-CR conditions [24,25]. This chronological extension by the CR diet was observed for two different aspects of the reproductive ability, including (1) the ability of a cell to form a colony after being transferred from a nutrient-depleted liquid medium to a surface of a nutrient-rich solid medium (Figure 2C) [24], and (2) the ability of a cell population to synchronously re-enter the mitotic cell cycle after being transferred from a nutrient-depleted liquid medium to a nutrient-rich liquid medium (Figure 2D) [24]. 

Glycogen and trehalose are the two major glucose storage molecules in *S. cerevisiae* [3,4,58]. Trehalose in budding yeast also contributes to the protection of the entire cell and its protein constituents from various stresses, maintenance of cellular proteostasis, and mitotic division of Q cells under non-CR conditions [59,60,61,62,63,64,65,66,67]. A substantial rise in the concentrations of glycogen and trehalose is a characteristic trait of Q and NQ cell sub-populations developed in yeast cultures that are limited in calorie supply (Figure 2E) [24,25]. Indeed, CR significantly increases the concentrations of glycogen and trehalose in both Q and NQ cells beginning of the PD phase of culturing (Figure 2E) [24]. Of note, glycogen and trehalose concentrations within Q cells formed in yeast cultures under CR conditions considerably exceed those within age-matched NQ cells developed in these cultures [24]. 

After the neutral lipids triacylglycerols (TAG) are synthesized in the endoplasmic reticulum (ER), they are stored in lipid droplets (LD) as the source of free (non-esterified) fatty acids for energy metabolism and phospholipid biosynthesis [68,69,70,71,72,73,74]. One of the characteristic traits of Q and NQ cell sub-populations formed in *S. cerevisiae* cultured under CR conditions is a considerable decline in TAG concentration, as compared to age-matched Q and NQ cell sub-populations developed in this yeast cultured under non-CR conditions (Figure 2F) [24,25]. Such a decline in TAG concentration is observed through the entire chronological lifespan [24]. Notably, there is no significant difference between age-matched Q and NQ cells regarding TAG concentration in these cells at any stage of the chronological aging process in *S. cerevisiae* [24]. 

Cardiolipins (CL) are signature lipids of the inner mitochondrial membrane essential for the maintenance of proper morphology and functionality of yeast mitochondria [68,75,76,77,78,79]. Mitochondria of both Q and NQ cell sub-populations in *S. cerevisiae* limited in calorie supply exhibit an increase in CL concentration beginning of the PD growth phase (Figure 2G) [24,25]. The increase in CL concentration is a characteristic trait of mitochondria present in Q and NQ cells under CR conditions, as compared to mitochondria found in Q and NQ cells (respectively) under non-CR conditions [24,25]. However, CL concentration in mitochondria of Q cells does not significantly differ from those in mitochondria of age-matched NQ cells regardless of the extent of calorie supply [24]. 

The rate of mitochondrial respiration and electrochemical potential across the inner mitochondrial membrane (ΔΨ_m_) are essential contributors to longevity assurance in chronologically aging *S. cerevisiae* [2,6,80,81,82,83,84,85,86]. CR substantially increases the rate of mitochondrial respiration and ΔΨ_m_ in both Q and NQ cells through the chronological lifespan of budding yeast (Figure 2H) [24,25]. The increase in these key aspects of mitochondrial functionality is one of the characteristic traits of Q and NQ cells limited in calorie supply, in comparison with Q and NQ cells (respectively) not limited in calorie supply [24,25]. Of note, the rate of respiration and ΔΨ_m_ in mitochondria of Q cells cultured under CR conditions exceed those in mitochondria of age-matched NQ cells in these cultures, especially during the ST growth phase [24]. 

An age-related dynamic of changes in cellular ROS concentrations within Q and NQ cells under CR conditions differs from the one within each of these cell sub-populations under non-CR conditions. Early in chronological lifespan, before the entry of cell culture into the ST growth phase, ROS concentrations within Q and NQ cells under CR conditions are significantly lower than those within age-matched Q and NQ cells (respectively) under non-CR conditions (Figure 2I) [24,25]. In contrast, late in chronological lifespan, before the entry of cell culture into the ST growth phase, ROS concentrations within Q and NQ cells under CR conditions are substantially higher than those within age-matched Q and NQ cells (respectively) under non-CR conditions (Figure 2I) [24,25]. The distinct age-related dynamic of changes in cellular ROS concentrations is a characteristic trait of Q and NQ cells of *S. cerevisiae* that are cultured under CR conditions. Of note, ROS are generated mostly as by-products of mitochondrial respiration [3,87] and play essential roles in regulating longevity of chronologically aging *S. cerevisiae* [2,6,84,85,88,89,90,91,92,93].

Age-related accumulation of oxidatively damaged cellular macromolecules contributes to the process of chronological aging in budding yeast [6,84,85,88,93,94,95]. One of the characteristic traits of Q and NQ cells in *S. cerevisiae* cultured under CR conditions is a significant decline in the extent of ROS-inflicted and age-related oxidative damage to proteins, lipids, nuclear DNA (nDNA), and mitochondrial DNA (mtDNA) (Figure 2J) [24,25]. A degree of such a CR-dependent decline in oxidative macromolecular damage varies for different macromolecules and stages of the chronological aging process [24]. Furthermore, the concentrations of all these oxidatively damaged macromolecules within Q cells limited in calorie supply are lower than within calorically restricted NQ cells of the same chronological age [24].

One of the characteristic traits of Q and NQ cells of *S. cerevisiae* cultured under CR conditions is a significant rise in the resistance to chronic (long-term) thermal and oxidative stresses, as compared to age-matched Q and NQ cells of this yeast cultured under non-CR conditions (Figure 2K) [24,25]. CR promotes the resistance of Q cells to both types of chronic stresses late in chronological lifespan, in the ST growth phase [24]. The stimulatory effect of CR on the tolerance of NQ cells to these stresses is observed early in chronological lifespan, in the PD growth phase [24]. Of note, the longevity of chronologically aging yeast can be extended by the interventions that enhance cell tolerance to chronic thermal and oxidative stresses [2,6,84,88,94,96,97,98,99,100]. 

Apoptotic and/or necrotic forms of RCD are hallmark processes taking place in chronologically “old” *S. cerevisiae* cells not limited in calorie supply [6,101,102,103,104,105,106,107,108,109,110,111,112]. CR postpones an age-related onset of apoptotic and necrotic RCD in both Q and NQ cell sub-populations (Figure 2L) [24,25]. In Q cells limited in calorie supply, a significant delay of the onsets of both forms of RCD occurs late in chronological lifespan in the ST growth phase [24]. In NQ cells of yeast cultures under CR conditions, the onsets of both forms of RCD are prolonged earlier in chronological lifespan, already when these cultures enter the PD growth phase [24]. Notably, the percentage of cells exhibiting characteristic traits of an apoptotic or necrotic form of RCD in NQ cell sub-populations from CR cultures exceeds that in age-matched Q cell sub-populations from these cultures [24]. 

CR not only delays an age-related onset of apoptotic and necrotic RCD in Q and NQ cells. This low-calorie diet also makes both cell sub-populations less susceptible to the exogenously induced apoptotic and necrotic forms of RCD (Figure 2M) [24,25]. The apoptotic form of RCD can be induced by a short-term treatment of yeast with exogenous hydrogen peroxide [102,105,110,113,114]. In contrast, the so-called “liponecrotic” form of RCD can occur in response to brief exposure of yeast to exogenous palmitoleic acid [109,110,111,112]. Q cells cultured under CR conditions become less susceptible to both forms of exogenously induced RCD than Q cells under non-CR conditions late in chronological lifespan, after the culture enters the ST growth phase [24]. Yet, NQ cells cultured under CR conditions exhibit lower susceptibility to both forms of exogenously induced RCD than NQ cells under non-CR conditions early life, after entry of the culture into the PD phase of culturing [24].

## 4. A Hypothesis: The CR Diet Slows Yeast Chronological Aging Because It Alters an Age-Related Chronology and Certain Properties of Q Cells

**Figure 3:**
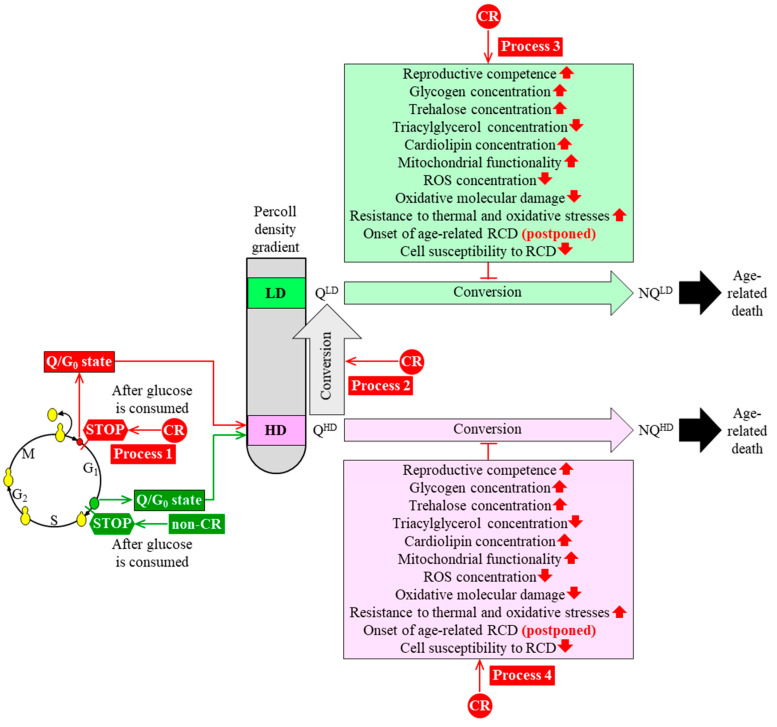
A hypothesis on how caloric restriction (CR) can slow chronological aging of budding yeast by altering the chronology and properties of quiescent (Q) cells. The hypothesis postulates that CR delays yeast chronological aging by targeting four processes within Q cells. See the text for more details. Other abbreviations: HD, high-density cells; LD, low-density cells; Q^HD^, quiescent cell of high density; QLD, quiescent cell of low density; NQHD, non-quiescent cell of high density; NQLD, non-quiescent cell of low density; RCD, regulated cell death; ROS, reactive oxygen species. 🡱 Increased by CR; 🡳 Decreased by CR; ˧ Slows the conversion of QLD cells into NQLD cells or the QHD-into-NQHD cell conversion.

We analyzed changes in an age-related chronology and various properties of Q and NQ cells that were purified from yeast populations cultured under CR or non-CR conditions. Our analysis suggests a hypothesis on how CR can slow yeast chronological aging by altering the chronology and properties of Q cells. This hypothesis is discussed below and schematically depicted in Figure 3.

Our hypothesis posits that CR slows yeast chronological aging by targeting the following four processes within Q cells.

First, because under CR conditions the cell cycle is arrested earlier in G_1_ than it is under non-CR conditions, high-density Q (Q^HD^) cells in CR cultures are significantly smaller than Q^HD^ cells in non-CR cultures (Figure 3, process 1) [24]. Future studies will need to examine if the ability of CR to create small Q^HD^ cells by arresting the cell cycle at a checkpoint in early G_1_ contributes to the ability of CR to delay yeast chronological aging. It is conceivable that some of the pro-longevity properties of Q^HD^ cells formed under CR conditions (such as an improved reproductive ability and/or other properties named in Figure 3) are due to the ability of CR to arrest the cell cycle in early G_1_, thus creating small Q^HD^ cells.

Second, CR speeds up an age-related conversion of Q^HD^ cells into (Q^LD^) cells in chronologically aging yeast cultures (Figure 3, process 2) [24,25]. A challenge for the future is to examine if the ability of CR to promote such conversion is an essential contributor to the CR-dependent delay of yeast chronological aging. It is plausible that a stimulating effect of CR on the Q^HD^-into-Q^LD^ cell conversion contributes to the pro-longevity properties of Q^LD^ cells formed under CR conditions. These properties of Q^LD^ cells might include their enhanced reproductive potential and/or other traits characteristic of Q^LD^ cells (Figure 3).

Third, a conversion of long-lived Q^LD^ cells into short-lived non-quiescent cells of low density (which we call NQ^LD^) under CR conditions occurs slower than under non-CR conditions (Figure 3, process 3) [24]. In our hypothesis, the ability of CR to decelerate the Q^LD^-into-NQ^LD^ cell conversion might contribute to the CR-dependent delay of yeast chronological aging because it allows Q^LD^ cells to maintain the pro-longevity cellular traits. These pro-longevity traits of Q^LD^ cells include an enhanced reproductive competence, increased glycogen and trehalose concentrations, a declined TAG concentration, an elevated concentration of CL, improved functionality of mitochondria, a reduced concentration of ROS, a decline in oxidative damage to macromolecules, postponed onsets of apoptotic and necrotic modes of RCD, and decreased susceptibilities to apoptotic and liponecrotic forms of RCD (Figure 3, process 3) [24].

Fourth, Q^HD^ cells formed under CR conditions maintain quiescence longer than Q^HD^ cells developed under non-CR conditions (Figure 3, process 4) [24]. Thus, CR slows the conversion of long-lived Q^HD^ cells into short-lived non-quiescent cells of high density (which we call NQ^HD^). According to our hypothesis, the CR-dependent deceleration of the Q^HD^-into-NQ^HD^ cell conversion might contribute to the delay of yeast chronological aging by this low-calorie diet because it allows Q^HD^ cells to sustain a pro-longevity cellular pattern. Such pro-longevity cellular pattern is maintained under CR conditions longer than under non-CR conditions because CR has similar effects on the pro-longevity cellular traits of Q^HD^ cells as the ones described above for Q^LD^ cells (Figure 3, process 4) [24].

## 5. Conclusions

In this review, we discussed mechanisms linking chronological aging to a program of cellular quiescence in the yeast *S. cerevisiae*. Our discussion indicates that the pace of yeast chronological aging depends on a complex program underlying cellular quiescence entry, maintenance, and exit. A CR diet remodels the cellular quiescence program, and such remodeling could contribute to the CR-dependent delay of yeast chronological aging. A challenge for the future is to define mechanisms by which the CR-driven remodeling of the cellular quiescence program could be linked to the CR-dependent delay of yeast chronological aging. Because the mechanisms of cellular aging and cellular quiescence have been conserved in the evolution, addressing these challenges in the future will increase our understanding of how the knowledge-based targeting of the cellular quiescence program can be used for delaying cellular and organismal aging and for postponing the onset of aging-associated diseases. 

## References

[1] Sinclair D.A. (2005). Toward a unified theory of caloric restriction and longevity regulation. Mech. Ageing Dev..

[2] Goldberg A.A., Bourque S.D., Kyryakov P., Gregg C., Boukh-Viner T., Beach A., Burstein M.T., Machkalyan G., Richard V., Rampersad S. (2009). Effect of calorie restriction on the metabolic history of chronologically aging yeast. Exp. Gerontol..

[3] Fraenkel D.G. (2011). Yeast Intermediary Metabolism.

[4] Broach J.R. (2012). Nutritional control of growth and development in yeast. Genetics.

[5] Longo V.D., Shadel G.S., Kaeberlein M., Kennedy B. (2012). Replicative and chronological aging in *Saccharomyces cerevisiae*. Cell Metab..

[6] Arlia-Ciommo A., Leonov A., Piano A., Svistkova V., Titorenko V.I. (2014). Cell-autonomous mechanisms of chronological aging in the yeast *Saccharomyces cerevisiae*. Microb. Cell.

[7] Allen C., Büttner S., Aragon A.D., Thomas J.A., Meirelles O., Jaetao J.E., Benn D., Ruby S.W., Veenhuis M., Madeo F. (2006). Isolation of quiescent and nonquiescent cells from yeast stationary-phase cultures. J. Cell Biol..

[8] Davidson G.S., Joe R.M., Roy S., Meirelles O., Allen C.P., Wilson M.R., Tapia P.H., Manzanilla E.E., Dodson A.E., Chakraborty S. (2011). The proteomics of quiescent and nonquiescent cell differentiation in yeast stationary-phase cultures. Mol. Biol. Cell.

[9] De Virgilio C. (2012). The essence of yeast quiescence. FEMS Microbiol. Rev..

[10] Werner-Washburne M., Roy S., Davidson G.S. (2012). Aging and the survival of quiescent and non-quiescent cells in yeast stationary-phase cultures. Subcell. Biochem..

[11] Miles S., Li L., Davison J., Breeden L.L. (2013). Xbp1 directs global repression of budding yeast transcription during the transition to quiescence and is important for the longevity and reversibility of the quiescent state. PLoS Genet..

[12] Aragon A.D., Rodriguez A.L., Meirelles O., Roy S., Davidson G.S., Tapia P.H., Allen C., Joe R., Benn D., Werner-Washburne M. (2008). Characterization of differentiated quiescent and nonquiescent cells in yeast stationary-phase cultures. Mol. Biol. Cell.

[13] Smets B., Ghillebert R., De Snijder P., Binda M., Swinnen E., De Virgilio C., Winderickx J. (2010). Life in the midst of scarcity: Adaptations to nutrient availability in *Saccharomyces cerevisiae*. Curr. Genet..

[14] Peters T.W., Rardin M.J., Czerwieniec G., Evani U.S., Reis-Rodrigues P., Lithgow G.J., Mooney S.D., Gibson B.W., Hughes R.E. (2012). Tor1 regulates protein solubility in *Saccharomyces cerevisiae*. Mol. Biol. Cell.

[15] Conrad M., Schothorst J., Kankipati H.N., Van Zeebroeck G., Rubio-Texeira M., Thevelein J.M. (2014). Nutrient sensing and signaling in the yeast *Saccharomyces cerevisiae*. FEMS Microbiol. Rev..

[16] Swinnen E., Ghillebert R., Wilms T., Winderickx J. (2014). Molecular mechanisms linking the evolutionary conserved TORC1-Sch9 nutrient signalling branch to lifespan regulation in *Saccharomyces cerevisiae*. FEMS Yeast Res..

[17] Sagot I., Laporte D. (2019). The cell biology of quiescent yeast—A diversity of individual scenarios. J. Cell Sci..

[18] Fontana L., Partridge L., Longo V.D. (2010). Extending healthy life span—From yeast to humans. Science.

[19] De Cabo R., Carmona-Gutierrez D., Bernier M., Hall M.N., Madeo F. (2014). The search for antiaging interventions: From elixirs to fasting regimens. Cell.

[20] Madeo F., Carmona-Gutierrez D., Hofer S.J., Kroemer G. (2019). Caloric restriction mimetics against age-associated disease: Targets, mechanisms, and therapeutic potential. Cell Metab..

[21] Weissman J., Guthrie C., Fink G.R. (2010). Guide to Yeast Genetics: Functional Genomics, Proteomics, and Other Systems Analysis.

[22] Botstein D., Fink G.R. (2011). Yeast: An experimental organism for 21st Century biology. Genetics.

[23] Feldmann H. (2012). Yeast: Molecular and Cell Biology.

[24] Leonov A., Feldman R., Piano A., Arlia-Ciommo A., Lutchman V., Ahmadi M., Elsaser S., Fakim H., Heshmati-Moghaddam M., Hussain A. (2017). Caloric restriction extends yeast chronological lifespan via a mechanism linking cellular aging to cell cycle regulation, maintenance of a quiescent state, entry into a non-quiescent state and survival in the non-quiescent state. Oncotarget.

[25] Mohammad K., Titorenko V.I. (2018). Yeast chronological aging is linked to cell cycle regulation. Cell Cycle.

[26] Chughtai Z.S., Rassadi R., Matusiewicz N., Stochaj U. (2001). Starvation promotes nuclear accumulation of the hsp70 Ssa4p in yeast cells. J. Biol. Chem..

[27] Narayanaswamy R., Levy M., Tsechansky M., Stovall G.M., O’Connell J.D., Mirrielees J., Ellington A.D., Marcotte E.M. (2009). Widespread reorganization of metabolic enzymes into reversible assemblies upon nutrient starvation. Proc. Natl. Acad. Sci. USA.

[28] Noree C., Sato B.K., Broyer R.M., Wilhelm J.E. (2010). Identification of novel filament-forming proteins in *Saccharomyces cerevisiae* and *Drosophila melanogaster*. J. Cell Biol..

[29] Tapia H., Morano K.A. (2010). Hsp90 nuclear accumulation in quiescence is linked to chaperone function and spore development in yeast. Mol. Biol. Cell.

[30] Liu I.C., Chiu S.W., Lee H.Y., Leu J.Y. (2012). The histone deacetylase Hos2 forms an Hsp42-dependent cytoplasmic granule in quiescent yeast cells. Mol. Biol. Cell.

[31] Noree C., Monfort E., Shiau A.K., Wilhelm J.E. (2014). Common regulatory control of CTP synthase enzyme activity and filament formation. Mol. Biol. Cell.

[32] Shah K.H., Nostramo R., Zhang B., Varia S.N., Klett B.M., Herman P.K. (2014). Protein kinases are associated with multiple, distinct cytoplasmic granules in quiescent yeast cells. Genetics.

[33] Taddei A., Schober H., Gasser S.M. (2010). The budding yeast nucleus. Cold Spring Harb. Perspect. Biol..

[34] Laporte D., Courtout F., Salin B., Ceschin J., Sagot I. (2013). An array of nuclear microtubules reorganizes the budding yeast nucleus during quiescence. J. Cell Biol..

[35] Laporte D., Sagot I. (2014). Microtubules move the nucleus to quiescence. Nucl. Austin Tex..

[36] Guidi M., Ruault M., Marbouty M., Loïodice I., Cournac A., Billaudeau C., Hocher A., Mozziconacci J., Koszul R., Taddei A. (2015). Spatial reorganization of telomeres in long-lived quiescent cells. Genome Biol..

[37] McKnight J.N., Boerma J.W., Breeden L.L., Tsukiyama T. (2015). Global promoter targeting of a conserved lysine deacetylase for transcriptional shutoff during quiescence entry. Mol. Cell.

[38] Rutledge M.T., Russo M., Belton J.M., Dekker J., Broach J.R. (2015). The yeast genome undergoes significant topological reorganization in quiescence. Nucleic Acids Res..

[39] Laporte D., Courtout F., Tollis S., Sagot I. (2016). Quiescent *Saccharomyces cerevisiae* forms telomere hyperclusters at the nuclear membrane vicinity through a multifaceted mechanism involving Esc1, the Sir complex, and chromatin condensation. Mol. Biol. Cell.

[40] Miles S., Breeden L. (2017). A common strategy for initiating the transition from proliferation to quiescence. Curr. Genet..

[41] Roche B., Arcangioli B., Martienssen R. (2017). Transcriptional reprogramming in cellular quiescence. RNA Biol..

[42] Ramachandran V., Shah K.H., Herman P.K. (2011). The cAMP-dependent protein kinase signaling pathway is a key regulator of P body foci formation. Mol. Cell.

[43] Shah K.H., Zhang B., Ramachandran V., Herman P.K. (2013). Processing body and stress granule assembly occur by independent and differentially regulated pathways in *Saccharomyces cerevisiae*. Genetics.

[44] Riback J.A., Katanski C.D., Kear-Scott J.L., Pilipenko E.V., Rojek A.E., Sosnick T.R., Drummond D.A. (2017). Stress-triggered phase separation is an adaptive, evolutionarily tuned response. Cell.

[45] Peters L.Z., Karmon O., Miodownik S., Ben-Aroya S. (2016). Proteasome storage granules are transiently associated with the insoluble protein deposit in *Saccharomyces cerevisiae*. J. Cell Sci..

[46] Laporte D., Salin B., Daignan-Fornier B., Sagot I. (2008). Reversible cytoplasmic localization of the proteasome in quiescent yeast cells. J. Cell Biol..

[47] Weberruss M.H., Savulescu A.F., Jando J., Bissinger T., Harel A., Glickman M.H., Enenkel C. (2013). Blm10 facilitates nuclear import of proteasome core particles. EMBO J..

[48] Gu Z.C., Wu E., Sailer C., Jando J., Styles E., Eisenkolb I., Kuschel M., Bitschar K., Wang X., Huang L. (2017). Ubiquitin orchestrates proteasome dynamics between proliferation and quiescence in yeast. Mol. Biol. Cell.

[49] Marshall R.S., Vierstra R.D. (2018). Proteasome storage granules protect proteasomes from autophagic degradation upon carbon starvation. eLife.

[50] Sagot I., Pinson B., Salin B., Daignan-Fornier B. (2006). Actin bodies in yeast quiescent cells: An immediately available actin reserve?. Mol. Biol. Cell.

[51] Laporte D., Gouleme L., Jimenez L., Khemiri I., Sagot I. (2018). Mitochondria reorganization upon proliferation arrest predicts individual yeast cell fate. eLife.

[52] Hartwell L.H., Unger M.W. (1977). Unequal division in *Saccharomyces cerevisiae* and its implications for the control of cell division. J. Cell Biol..

[53] Powell C.D., Quain D.E., Smart K.A. (2003). Chitin scar breaks in aged *Saccharomyces cerevisiae*. Microbiology.

[54] Cabib E., Arroyo J. (2013). How carbohydrates sculpt cells: Chemical control of morphogenesis in the yeast cell wall. Nat. Rev. Microbiol..

[55] Barton A.A. (1950). Some aspects of cell division in *Saccharomyces cerevisiae*. J. Gen. Microbiol..

[56] Egilmez N.K., Chen J.B., Jazwinski S.M. (1990). Preparation and partial characterization of old yeast cells. J. Gerontol..

[57] Sinclair D., Mills K., Guarente L. (1998). Aging in *Saccharomyces cerevisiae*. Annu. Rev. Microbiol..

[58] François J., Parrou J.L. (2001). Reserve carbohydrates metabolism in the yeast *Saccharomyces cerevisiae*. FEMS Microbiol. Rev..

[59] Singer M.A., Lindquist S. (1998). Multiple effects of trehalose on protein folding in vitro and in vivo. Mol. Cell.

[60] Singer M.A., Lindquist S. (1998). Thermotolerance in *Saccharomyces cerevisiae*: The Yin and Yang of trehalose. Trends Biotechnol..

[61] Benaroudj N., Lee D.H., Goldberg A.L. (2001). Trehalose accumulation during cellular stress protects cells and cellular proteins from damage by oxygen radicals. J. Biol. Chem..

[62] Gancedo C., Flores C.L. (2004). The importance of a functional trehalose biosynthetic pathway for the life of yeasts and fungi. FEMS Yeast Res..

[63] Shi L., Sutter B.M., Ye X., Tu B.P. (2010). Trehalose is a key determinant of the quiescent metabolic state that fuels cell cycle progression upon return to growth. Mol. Biol. Cell.

[64] Trevisol E.T., Panek A.D., Mannarino S.C., Eleutherio E.C. (2011). The effect of trehalose on the fermentation performance of aged cells of *Saccharomyces cerevisiae*. Appl. Microbiol. Biotechnol..

[65] Kyryakov P., Beach A., Richard V.R., Burstein M.T., Leonov A., Levy S., Titorenko V.I. (2012). Caloric restriction extends yeast chronological lifespan by altering a pattern of age-related changes in trehalose concentration. Front. Physiol..

[66] Eleutherio E., Panek A., De Mesquita J.F., Trevisol E., Magalhães R. (2015). Revisiting yeast trehalose metabolism. Curr. Genet..

[67] Babazadeh R., Lahtvee P.J., Adiels C.B., Goksör M., Nielsen J.B., Hohmann S. (2017). The yeast osmostress response is carbon source dependent. Sci. Rep..

[68] Mitrofanova D., Dakik P., McAuley M., Medkour Y., Mohammad K., Titorenko V.I. (2018). Lipid metabolism and transport define the longevity of the yeast *Saccharomyces cerevisiae*. Front. Biosci. (Landmark Ed.).

[69] Walther T.C., Farese R.V. (2012). Lipid droplets and cellular lipid metabolism. Annu. Rev. Biochem..

[70] Kohlwein S.D., Veenhuis M., van der Klei I.J. (2013). Lipid droplets and peroxisomes: Key players in cellular lipid homeostasis or a matter of fat—Store ‘em up or burn ‘em down. Genetics.

[71] Pol A., Gross S.P., Parton R.G. (2014). Review: Biogenesis of the multifunctional lipid droplet: Lipids, proteins, and sites. J. Cell Biol..

[72] Gao Q., Goodman J.M. (2015). The lipid droplet—A well-connected organelle. Front. Cell. Dev. Biol..

[73] Walther T.C., Chung J., Farese R.V. (2017). Lipid droplet biogenesis. Annu. Rev. Cell. Dev. Biol..

[74] Jackson C.L. (2019). Lipid droplet biogenesis. Curr. Opin. Cell Biol..

[75] Horvath S.E., Daum G. (2013). Lipids of mitochondria. Prog. Lipid Res..

[76] Baile M.G., Lu Y.W., Claypool S.M. (2014). The topology and regulation of cardiolipin biosynthesis and remodeling in yeast. Chem. Phys. Lipids.

[77] Mårtensson C.U., Doan K.N., Becker T. (2017). Effects of lipids on mitochondrial functions. Biochim. Biophys. Acta.

[78] Schlame M., Greenberg M.L. (2017). Biosynthesis, remodeling and turnover of mitochondrial cardiolipin. Biochim. Biophys. Acta Mol. Cell Biol. Lipids.

[79] Tatsuta T., Langer T. (2017). Intramitochondrial phospholipid trafficking. Biochim. Biophys. Acta.

[80] Bonawitz N.D., Chatenay-Lapointe M., Pan Y., Shadel G.S. (2007). Reduced TOR signaling extends chronological life span via increased respiration and upregulation of mitochondrial gene expression. Cell Metab..

[81] Pan Y., Shadel G.S. (2009). Extension of chronological life span by reduced TOR signaling requires down-regulation of Sch9p and involves increased mitochondrial OXPHOS complex density. Aging (Albany N. Y.).

[82] Breitenbach M., Laun P., Dickinson J.R., Klocker A., Rinnerthaler M., Dawes I.W., Aung-Htut M.T., Breitenbach-Koller L., Caballero A., Nyström T. (2012). The role of mitochondria in the aging processes of yeast. Subcell. Biochem..

[83] Ocampo A., Liu J., Schroeder E.A., Shadel G.S., Barrientos A. (2012). Mitochondrial respiratory thresholds regulate yeast chronological life span and its extension by caloric restriction. Cell Metab..

[84] Leonov A., Titorenko V.I. (2013). A network of interorganellar communications underlies cellular aging. IUBMB Life.

[85] Beach A., Leonov A., Arlia-Ciommo A., Svistkova V., Lutchman V., Titorenko V.I. (2015). Mechanisms by which different functional states of mitochondria define yeast longevity. Int. J. Mol. Sci..

[86] Ruetenik A., Barrientos A. (2015). Dietary restriction, mitochondrial function and aging: From yeast to humans. Biochim. Biophys. Acta.

[87] Giorgio M., Trinei M., Migliaccio E., Pelicci P.G. (2007). Hydrogen peroxide: A metabolic by-product or a common mediator of ageing signals?. Nat. Rev. Mol. Cell Biol..

[88] Dakik P., Medkour Y., Mohammad K., Titorenko V.I. (2019). Mechanisms through which some mitochondria-generated metabolites act as second messengers that are essential contributors to the aging process in eukaryotes across phyla. Front. Physiol..

[89] Pan Y. (2011). Mitochondria, reactive oxygen species, and chronological aging: A message from yeast. Exp. Gerontol..

[90] Pan Y., Schroeder E.A., Ocampo A., Barrientos A., Shadel G.S. (2011). Regulation of yeast chronological life span by TORC1 via adaptive mitochondrial ROS signaling. Cell Metab..

[91] Shadel G.S. (2014). Live longer on MARS: A yeast paradigm of mitochondrial adaptive ROS signaling in aging. Microb. Cell.

[92] Guaragnella N., Coyne L.P., Chen X.J., Giannattasio S. (2018). Mitochondria-cytosol-nucleus crosstalk: Learning from *Saccharomyces cerevisiae*. FEMS Yeast Res..

[93] Ruetenik A., Barrientos A. (2018). Exploiting Post-Mitotic Yeast Cultures to Model Neurodegeneration. Front. Mol. Neurosci..

[94] Gladyshev V.N. (2013). The origin of aging: Imperfectness-driven non-random damage defines the aging process and control of lifespan. Trends Genet..

[95] Ogrodnik M., Salmonowicz H., Gladyshev V.N. (2019). Integrating cellular senescence with the concept of damage accumulation in aging: Relevance for clearance of senescent cells. Aging Cell.

[96] Calabrese V., Cornelius C., Cuzzocrea S., Iavicoli I., Rizzarelli E., Calabrese E.J. (2011). Hormesis, cellular stress response and vitagenes as critical determinants in aging and longevity. Mol. Asp. Med..

[97] Calabrese V., Cornelius C., Dinkova-Kostova A.T., Iavicoli I., Di Paola R., Koverech A., Cuzzocrea S., Rizzarelli E., Calabrese E.J. (2012). Cellular stress responses, hormetic phytochemicals and vitagenes in aging and longevity. Biochim. Biophys. Acta.

[98] Gladyshev V.N. (2014). The free radical theory of aging is dead. Long live the damage theory!. Antioxid. Redox Signal..

[99] Dakik P., Titorenko V.I. (2016). Communications between Mitochondria, the Nucleus, Vacuoles, Peroxisomes, the Endoplasmic Reticulum, the Plasma Membrane, Lipid Droplets, and the Cytosol during Yeast Chronological Aging. Front. Genet..

[100] Gomez-Perez A., Kyryakov P., Burstein M.T., Asbah N., Noohi F., Iouk T., Titorenko V.I. (2016). Empirical validation of a hypothesis of the hormetic selective forces driving the evolution of longevity regulation mechanisms. Front. Genet..

[101] Herker E., Jungwirth H., Lehmann K.A., Maldener C., Fröhlich K.U., Wissing S., Büttner S., Fehr M., Sigrist S., Madeo F. (2004). Chronological aging leads to apoptosis in yeast. J. Cell Biol..

[102] Büttner S., Eisenberg T., Herker E., Carmona-Gutierrez D., Kroemer G., Madeo F. (2006). Why yeast cells can undergo apoptosis: Death in times of peace, love, and war. J. Cell Biol..

[103] Fabrizio P., Longo V.D. (2008). Chronological aging-induced apoptosis in yeast. Biochim. Biophys. Acta.

[104] Eisenberg T., Knauer H., Schauer A., Büttner S., Ruckenstuhl C., Carmona-Gutierrez D., Ring J., Schroeder S., Magnes C., Antonacci L. (2009). Induction of autophagy by spermidine promotes longevity. Nat. Cell Biol..

[105] Carmona-Gutierrez D., Eisenberg T., Büttner S., Meisinger C., Kroemer G., Madeo F. (2010). Apoptosis in yeast: Triggers, pathways, subroutines. Cell Death Differ..

[106] Eisenberg T., Carmona-Gutierrez D., Büttner S., Tavernarakis N., Madeo F. (2010). Necrosis in yeast. Apoptosis.

[107] Laun P., Büttner S., Rinnerthaler M., Burhans W.C., Breitenbach M. (2012). Yeast aging and apoptosis. Subcell. Biochem..

[108] Eisenberg T., Büttner S. (2014). Lipids and cell death in yeast. FEMS Yeast Res..

[109] Richard V.R., Beach A., Piano A., Leonov A., Feldman R., Burstein M.T., Kyryakov P., Gomez-Perez A., Arlia-Ciommo A., Baptista S. (2014). Mechanism of liponecrosis, a distinct mode of programmed cell death. Cell Cycle.

[110] Sheibani S., Richard V.R., Beach A., Leonov A., Feldman R., Mattie S., Khelghatybana L., Piano A., Greenwood M., Vali H. (2014). Macromitophagy, neutral lipids synthesis and peroxisomal fatty acid oxidation protect yeast from “liponecrosis,” a previously unknown form of programmed cell death. Cell Cycle.

[111] Arlia-Ciommo A., Svistkova V., Mohtashami S., Titorenko V.I. (2016). A novel approach to the discovery of anti-tumor pharmaceuticals: Searching for activators of liponecrosis. Oncotarget.

[112] Falcone C., Mazzoni C. (2016). External and internal triggers of cell death in yeast. Cell. Mol. Life Sci..

[113] Fannjiang Y., Cheng W.C., Lee S.J., Qi B., Pevsner J., McCaffery J.M., Hill R.B., Basañez G., Hardwick J.M. (2004). Mitochondrial fission proteins regulate programmed cell death in yeast. Genes Dev..

[114] Pereira C., Silva R.D., Saraiva L., Johansson B., Sousa M.J., Côrte-Real M. (2008). Mitochondria-dependent apoptosis in yeast. Biochim. Biophys. Acta.

